# P-322. Transmission of Methicillin-Resistant *Staphylococcus aureus* to Healthcare Personnel Gloves and Gowns: a Cohort Study of VA Hospitals in 5 States

**DOI:** 10.1093/ofid/ofae631.525

**Published:** 2025-01-29

**Authors:** Lyndsay M O’Hara, Michelle Newman, Lisa Pineles, Michael Rubin, Karim Khader, Richard Nelson, Mary Bahr-Robertson, Gio Baracco, Matthew B Goetz, Martin Evans, Kathleen A Linder, Eli N Perencevich, Anthony Harris

**Affiliations:** University of Maryland School of Medicine, Baltimore, Maryland; University of Maryland Baltimore, Baltimore, Maryland; University of Maryland School of Medicine, Baltimore, Maryland; University of Utah, Salt Lake City, Utah; University of Utah, Salt Lake City, Utah; University of Utah, Salt Lake City, Utah; University of Maryland Baltimore, Baltimore, Maryland; Miami VA Healthcare System, Miami, Florida; VA Greater Los Angeles Healthcare System, Los Angeles, California; Veterans Affairs, Lexington, KY; University of Michigan/Ann Arbor VAMC, Ann Arbor, Michigan; University of Iowa/Iowa City VAMC, Iowa City, Iowa; University of Maryland School of Medicine, Baltimore, Maryland

## Abstract

**Background:**

The aim of this study was to determine the rate of MRSA transmission to healthcare personnel (HCP) gloves and gown and to determine which patient care interactions and HCP roles are associated with greater transmission of MRSA.

**Figure:**
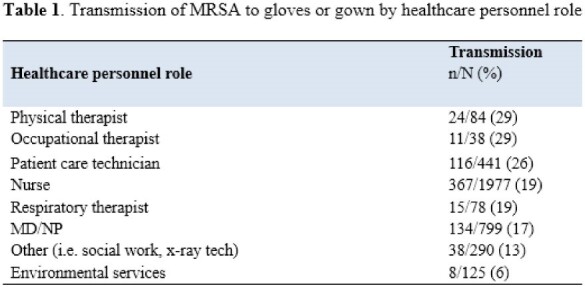

**Methods:**

Patients with a positive MRSA clinical or surveillance culture within the past 7 days were enrolled at 5 VA medical centers. Research team members observed 5 HCP in the room for each patient and recorded any items that were touched on a standardized data collection form. After completion of tasks and prior to room exit, HCP gloves and gown were cultured separately. The primary outcome was a surrogate measure for transmission defined as “yes” if a HCP glove or gown was contaminated with MRSA.

**Figure:**
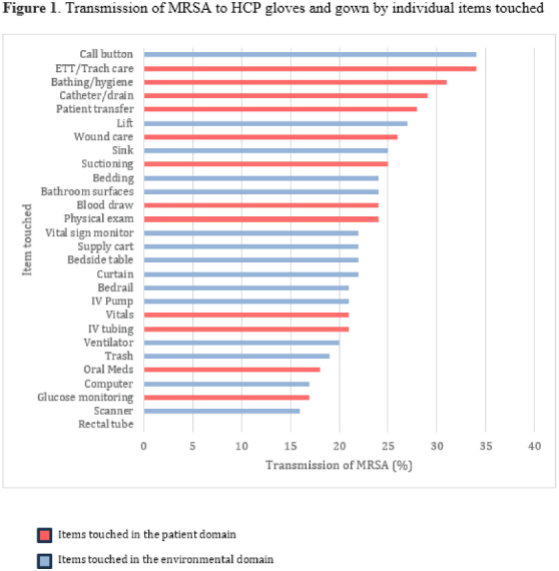

**Results:**

We enrolled 799 patients and obtained 3,832 glove and gown cultures. Transmission of MRSA to gloves or gown occurred 713/3832 (18.6%) of the time while 589/3832 (15.4%) of interactions resulted in contamination of gloves and 319/3831 (8.3%) of interactions resulted in contamination of gowns. The gloves and gown of physical therapists and occupational therapists were most frequently contaminated with transmission occurring in 24/84 (29%) and 11/38 (29%) of observations respectively (Table 1). Any interactions that involved touching the patient (patient alone or patient and environment) resulted in glove or gown contamination in 622/2901 (21.4%) of observations, while touching only the environment resulted contamination in 91/931 (9.8%) of observations. Specific patient care activities with the highest rates of transmission included touching the endotracheal tube (12/35, 34%) and assisting the patient with bathing and hygiene (89/283, 31%). Specific environmental items touched with the highest rates of transmission included the call button (75/221, 34%) and the lift (31/125, 27%) (Figure 1).

**Conclusion:**

Contamination of HCP gloves and gowns with MRSA occurs frequently when caring for Veteran patients particularly when there is direct patient contact. Hospitals may consider optimizing contact precautions by using fewer precautions for low-risk interactions and more precautions for high-risk interactions.

**Disclosures:**

**Anthony Harris, MD, MPH**, Innoviva: Advisor/Consultant|UpToDate: Infection Control Editor

